# Monocytes subsets altered distribution and dysregulated plasma hsa-miR-21-5p and hsa-miR-155-5p in HCV-linked liver cirrhosis progression to hepatocellular carcinoma

**DOI:** 10.1007/s00432-023-05313-w

**Published:** 2023-08-28

**Authors:** Reham Hammad, Mona A. Eldosoky, Asmaa A. Elmadbouly, Reda Badr Aglan, Sherihan G. AbdelHamid, Samy Zaky, Elham Ali, Fatma El-Zahraa Abd El Hakam, Alshaimaa M. Mosaad, Neamat A. Abdelmageed, Fatma M. Kotb, Hend G. Kotb, Ahmed A. Hady, Omaima I. Abo-Elkheir, Sandy Kujumdshiev, Ulrich Sack, Claude Lambert, Nadia M. Hamdy

**Affiliations:** 1 Clinical Pathology Department, Faculty of Medicine (Girls), Al-Azhar University, Nasr City, Cairo, 11884 Egypt; 2 Hepatology and Gastroenterology Department, National Liver Institute, Menoufia University, Shibîn el Kôm, 35211 Menoufia Egypt; 3 Biochemistry Department, Faculty of Pharmacy, Ain Shams University, Abbasia, Cairo, 11566 Egypt; 4 Hepatology, Gastroenterology and Infectious Diseases Department, Faculty of Medicine (Girls), Al-Azhar University, Nasr City, Cairo, 11884 Egypt; 5 Molecular Biology, Zoology and Entomology Department, Faculty of Science (Girls), Al-Azhar University, Nasr City, Cairo, 11754 Egypt; 6 Pharmacology Department, Faculty of Medicine (Girls), Al-Azhar University, Nasr City, Cairo, 11884 Egypt; 7 Internal Medicine Department, Faculty of Medicine (Girls), Al-Azhar University, Nasr City, Cairo, 11884 Egypt; 8 Clinical Oncology and Nuclear Medicine Department, Faculty of Medicine, Mansoura University, Mansoura, Egypt; 9 Community Medicine and Public Health Department, Faculty of Medicine (Girls), Al-Azhar University, Nasr City, Cairo, 11884 Egypt; 10 Institute of Clinical Immunology, University Medical Center Leipzig, Johannisallee 30, 04103 Leipzig, Germany; 11 DHGS German University of Health and Sport, Berlin, Germany; 12 Cytometry Unit, Immunology Laboratory, Saint-Etienne University Hospital, Saint-Étienne, Lyon, France

**Keywords:** HCC, hsa-miR-21-5p, hsa-miR-155-5p, Liver cirrhosis, Monocyte subsets, In silico analysis

## Abstract

**Purpose:**

The authors aim to investigate the altered monocytes subsets distribution in liver cirrhosis (LC) and subsequent hepatocellular carcinoma (HCC) in association with the expression level of plasma Homo sapiens (has)-miR-21-5p and hsa-miR-155-5p. A step toward non-protein coding (nc) RNA precision medicine based on the immune perturbation manifested as altered monocytes distribution, on top of LC and HCC.

**Methods:**

Seventy-nine patients diagnosed with chronic hepatitis C virus (CHCV) infection with LC were enrolled in the current study. Patients were sub-classified into LC group without HCC (*n* = 40), LC with HCC (*n* = 39), and 15 apparently healthy controls. Monocyte subsets frequencies were assessed by flow cytometry. Real-time quantitative PCR was used to measure plasma hsa-miR-21-5p and hsa-miR-155-5p expression.

**Results:**

Hsa-miR-21-5p correlated with intermediate monocytes (*r* = 0.30, *p* = 0.007), while hsa-miR-155-5p negatively correlated with non-classical monocytes (*r* = − 0.316, *p* = 0.005). ROC curve analysis revealed that combining intermediate monocytes frequency and hsa-miR-21 yielded sensitivity = 79.5%, specificity = 75%, and AUC = 0.84. In comparison, AFP yielded a lower sensitivity = 69% and 100% specificity with AUC = 0.85. Logistic regression analysis proved that up-regulation of intermediate monocytes frequency and hsa-miR-21-5p were independent risk factors for LC progression to HCC, after adjustment for co-founders.

**Conclusion:**

Monocyte subsets differentiation in HCC was linked to hsa-miR-21-5p and hsa-miR-155-5p. Combined up-regulation of intermediate monocytes frequency and hsa-miR-21-5p expression could be considered a sensitive indicator of LC progression to HCC. Circulating intermediate monocytes and hsa-miR-21-5p were independent risk factors for HCC evolution, clinically and in silico proved.

**Graphical abstract:**

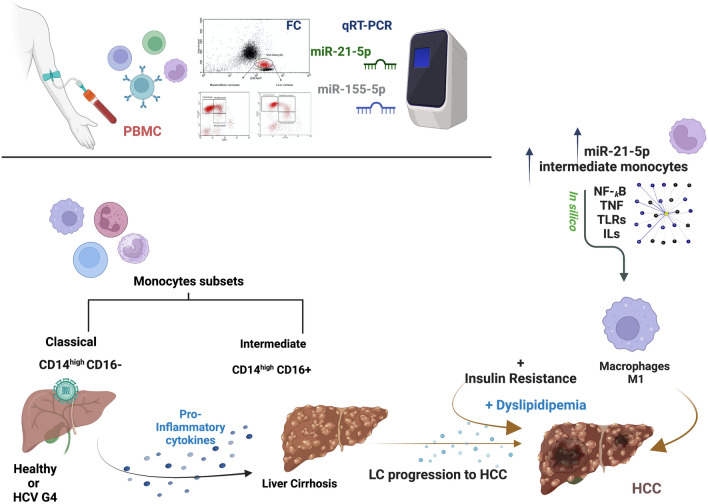

**Supplementary Information:**

The online version contains supplementary material available at 10.1007/s00432-023-05313-w.

## Introduction

Innate immunity activation and inflammation play key roles in liver disease development (Riva and Mehta 2019). The dynamic spectrum of immunological perturbations that develop in cirrhotic patients is referred to as cirrhosis-associated immune dysfunction (CAID) (Albillos et al. 2022). This starts with systemic inflammation exacerbating clinical manifestations of cirrhosis, followed by immunodeficiency (Irvine et al. 2019). The intensity of CAID has important consequences on cirrhosis progression and correlates with the severity of liver insufficiency and organ failure (Albillos et al. 2022). Innate immune cells, in particular monocytes, are pivotal effector and target cells in CAID (China et al. 2018). Circulating monocytes as they move through the liver contribute to the generation of damage-associated molecular patterns, which act as continual inflammatory stimuli, causing systemic perturbations and the release of inflammatory cytokines (Irvine et al. 2019). Damage-associated molecular patterns (DAMPs) drive the progression of cirrhosis via perpetuating inflammation (Krenkel and Tacke 2017).

Liver disease is thought to be responsible for around 2 million deaths worldwide (Maini et al. 2021). Hepatitis C virus (HCV) is a human hepatotropic pathogen that infects 58 million people globally, with a high mortality rate reaching 290,000 deaths annually (Joharji et al. 2022). In half of the cases, patients fail to clear the virus spontaneously and acute HCV infection progresses to chronic hepatitis C virus (CHCV) (Verstegen et al. 2015). CHCV infection could prompt liver cirrhosis (LC) and hepatocellular carcinoma (HCC) (El-Hefny et al. 2019). HCC normally develops in the setting of cirrhosis and the process of tumorigenesis is further promoted by HCV infection (Khare et al. 2022). In Egypt, about 80% of the patients with HCC have underling CHCV (Demerdash et al. 2017).

HCC prognosis varies greatly according to tumor stage at the time of diagnosis, so identifying cirrhotic HCC during liver cirrhosis stage is pivotal for improving the clinical outcomes of cirrhotic HCC patients (Caviglia et al. 2020).

MicroRNAs (miRs) are non-protein coding RNAs which play a vital role in regulating gene expression at various levels of transcription, translation, and protein function (Wei et al. 2019). Disturbed expression of miRs has been associated with the clinicopathological features of cirrhosis (Segarra et al. 2016), and development of HCC (Gupta et al. 2022). Few miRs have been regarded as master immune regulators of multiple cellular processes in HCC (Lu et al. 2022).

Homo sapiens (hsa)-miR-155-5p is a crucial regulator that controls cellular pro-inflammatory activities (Guo et al. 2020), and has been involved in both HCC and CHCV (Mohamed et al. 2020). Hsa-miR-21-5p is also a key molecular marker regulating different immune networks (Shu et al. 2022), and its over-expression in plasma was shown to have a potential value as a screening marker for HCC (Zhang et al. 2020). In our recent work, we found that both hsa-miR-21-5p and hsa-miR-155-5p plasma levels were shown to be related to the progression of LC to HCC, and showed potential diagnostic value in patients without elevated alpha-feto protein (AFP) (Eldosoky et al. 2023).

Monocytes subsets have different functional characteristics and roles during inflammation and/or malignancy (Yousef et al. 2020). The monocyte cluster of differentiation 14 (CD14) (Wolf et al. 2019) is identified by Uniport and GeneCards GeneCards^®^ (RRID:SCR_002773) in silico databases. CD14 is involved in mediating the innate immune response, on chromosome 5 reverse strand, activating the nuclear factor kappa-B cell (NF-_*K*_B), few cytokine secretions and the inflammatory response (Faure-Dupuy et al. 2017), as identified via the curated database *SIGnaling Network Open Resource*; Signor3.0 (Pubmed ID: 31665520) (Lo Surdo et al. 2023).

Fc gamma receptor IIIa (FCGR3A: FcγRIII) or CD16, on chromosome 1 reverse strand (Georg et al. 2022), is expressed on some monocytes surface, but, is more related to natural killer (NK) cells within tissues (Dogra et al. 2020), as identified by Gene-NCBI and the *Human Universal Single Cell Hub* (HUSCH) database using UMAP (RRID:SCR_018217) (Shi et al. 2023).

Peripheral blood monocytes are sub-classified according to expression of CD14 and CD16 into three subsets. The first subset is classical monocytes (CD14^high^ CD16−), that accounts for most of circulating monocytes in healthy individuals (Ong et al. 2019). This population has been reported to increase in cases of acute inflammation and is rapidly recruited to the infection scene (Shikuma et al. 2014). On the other hand, 5–10% of total blood monocytes express CD16 and are referred to as intermediate monocytes (CD14^high^ CD16+) which are potent producers of pro-inflammatory cytokines (Ruiz-Alcaraz et al. 2016), coming next are the non-classical monocytes (CD14^dim^ CD16^high^) (Coillard and Segura 2019; Gómez-olarte et al. 2019). This would support our potential interest in blood monocytes for monitoring LC development and its progression to HCC. In the same line, our recent research revealed an alteration of intermediate monocytes subset in LC and HCC (Ali et al. 2022).

In the setting of LC due to CHCV genotype 4 (G4) infection, and subsequent HCC, the relationship between intermediate monocytes and immune-regulatory miRs, hsa-miR-21-5p and hsa-miR-155-5p, coincidence remains to be examined.

Therefore, we aimed to investigate the clinical relevance of peripheral blood monocytes subsets distribution and circulating hsa-miR-21-5p and hsa-miR-155-5p in the development of LC-linked to CHCV G4 infection, as well as their role in LC progression to HCC. In addition, we aimed to perform an in silico databases search to provide an insight on immune cells in blood and liver as well as monocyte surface-CDs activation drivers. Second, using curated databases and text-mining to identify monocytes surface antigens interacting genes and their down-stream target genes, and monocytes surface antigens targeting genes.

## Subjects and methods

### Sample size and the study power

Based on the previous studies by Gu et al. (2021) and Hammad et al. (2022), sample size estimation was performed using the G power* sample size online calculator https://riskcalc.org/samplesize/# depending on a two-sided significance level of 0.05 and power (1 − beta) of 0.95. Estimated sample size was minimum of 40 patients’ vs 15 controls to reject the null hypothesis (power) of 0.9.

### Study participants

This study enrolled 79 patients with CHCV-related LC divided into Group 1, with early HCC (*n* = 39) and Group 2, without HCC (*n* = 40). These groups were compared to apparently healthy subjects (Group 3, *n* = 15).

Patients were recruited from the National Liver Institute, Menoufia University, Menoufia, Egypt, and Al-Zahraa University Hospital, Al-Azhar University, Cairo, Egypt. Patients’ Inclusion criteria: Child Pugh scores were used to categorize cirrhotic patients (Tsoris and Marlar 2023). A blind abdominal computed tomography (CT) scan was performed using Siemens 128 (Germany). CHCV fulfilling imaging criteria in accordance with recent recommendations were used to confirm the HCC diagnosis. The Barcelona Clinic Liver Cancer (BCLC) classification system was used to stage HCC patients (Tellapuri et al. 2018).

### Participants’ assessment

Age, gender, and medical history were retrieved (after ethical approval) from the hospital medical records. Participants underwent general clinical examination and assessment of their body mass index (BMI). The control group included age and sex-matched apparently healthy blood donors in Al-Zahraa University Hospital Blood Bank, who were informed and asked to join the study. They were enrolled only after negative viral hepatitis screening and normal results were reported from check-up laboratory tests.

### Patients’ exclusion criteria

Patients with a history of alcoholism or autoimmune disease, acute or chronic HBV (as determined by serology), HCC not mediated by CHCV, and patients who were undergoing any type of radiation or chemotherapy for a malignancy other than HCC were excluded.

### Blood sampling

Peripheral blood samples (6 mL) were collected. Blood was divided into 3 tubes. First EDTA tube was used for complete blood count and the flow cytometry (FC) assay. Second EDTA tube was centrifuged for 10 min at 1900×*g*, after which the plasma was carefully withdrawn and centrifuged again for 10 min at 16,000×*g* at 4 °C to remove additional cellular nucleic acids attached to cell debris. The supernatant was then transferred to micro-centrifuge vials and stored at − 80 °C until miRNA extraction, and third yellow-capped tube was used for serum separation routine lab analysis.

### Assays and analysis

#### Flow cytometry assay

**Fig. 1:**
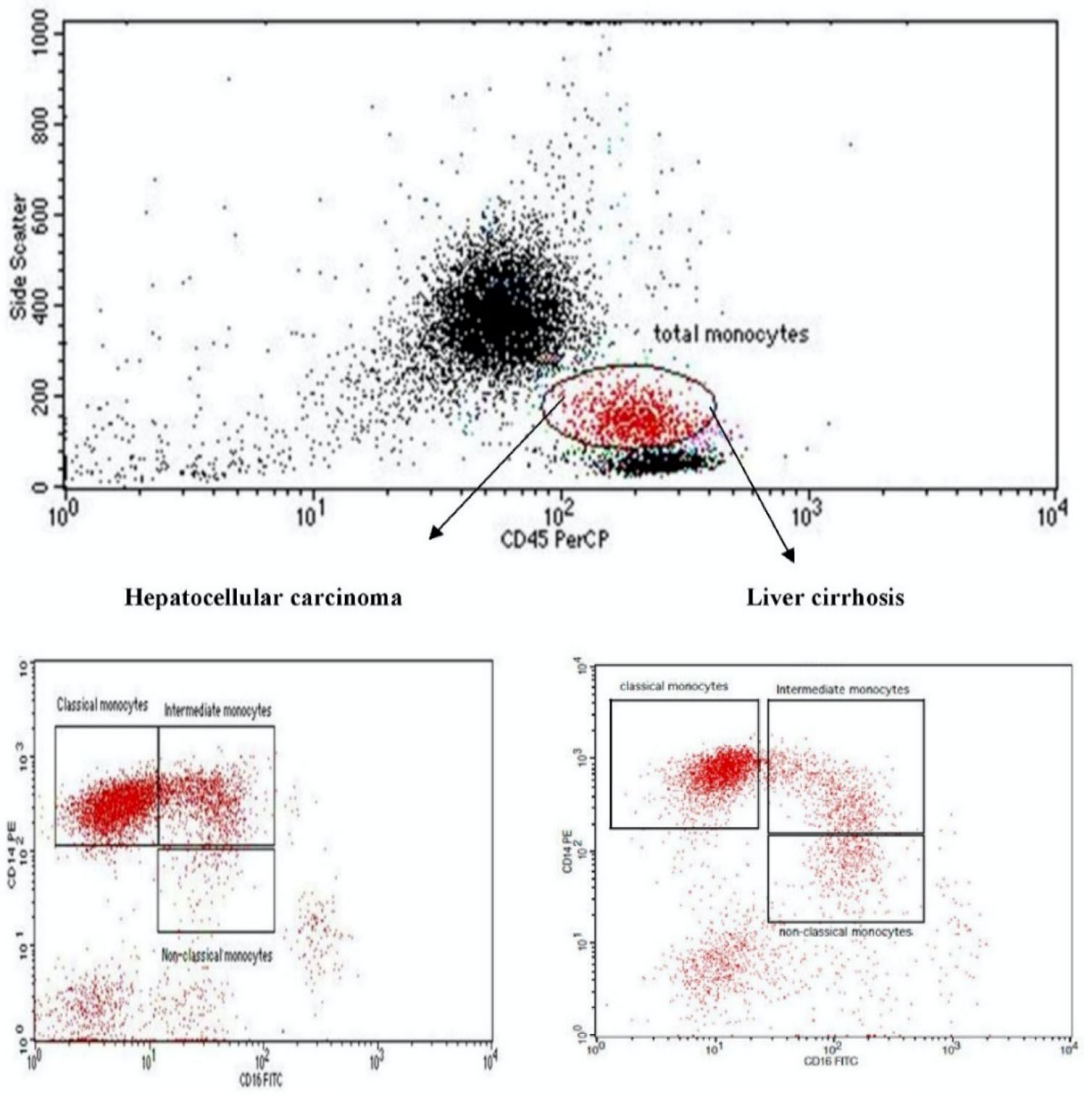
Gating strategy for detection of monocytes subsets in HCC group (lower left) and liver cirrhosis group (lower right); the initial gate was taken on dot-plot graph using forward scatter (FS)/CD45-PerCP and total monocytes-were defined (upper graph). On quadrant plot using CD14-PE (*y*-axis) and CD16-FITC (*x* axis) for determination of classical (CD14^high^ CD16−), intermediate (CD14^high^ CD16+), and non-classical monocytes subsets (CD14^dim^ CD16+). An example of an HCC patient and LC patient is displayed

FACSCalibur (Biosciences, San Jose, USA) was used to investigate the different phenotypes of monocyte subsets. A volume of 50 µL blood was incubated for 20 min with 5 µL CD14-PE-conjugated Ab (cat. no. A07764, lot. no.25, BD Biosciences) and 5 µL CD16-FITC-conjugated Ab (cat. no. P59232AA, lot no.200105, Immunotech; Beckman Coulter, Marseille, France), and 5 µL CD45-PerCP-conjugated anti-human (cat. no. 345809, lot no. 6039924, BD Biosciences, USA). Red blood cells were lysed. Samples were washed and suspended in phosphate buffer saline. The initial gate was taken on dot-plot graph using side scatter (SS)/CD45-PerCP and total monocytes area was defined. Monocytes subsets were defined on another quadrant plot, using CD14-PE and CD16-FITC. Percentage of monocytes subsets was determined from the total monocyte gate, and accordingly, the frequency of the monocytes subsets from the total events acquired was detected. The gating strategy is following the steps of our previous publication Ali et al. (2022). Gating strategy and an example of each patients group are displayed in Fig. 1.

#### MiRNA extraction and qRT-PCR

Mature plasma hsa-miR-21-5p and hsa-miR-155-5p were extracted from 200 µL of stored plasma using miRNeasy commercial kit (Cat. NO. 217004, Qiagen, Germany), according to the manufacturer’s protocol. Purity of extracted RNA was tested spectrophotometrically at 260/280 nm. Synthesis of complementary DNA (cDNA) was carried out using miRCURY LNA RT Kit (Cat. No. 339340, Qiagen, Germany) according to the manufacturer’s instructions. Hsa-miR-21-5p and hsa-miR-155-5p expression were determined using miRCURY LNA SYBR Green PCR Kit (Cat. No. 339345, Qiagen, Germany), following manufacturer’s protocol, utilizing a real-time PCR quaint studio 5 system (Applied Biosystem, USA). An internal housekeeping endogenous control, miR SNORD68, was employed. The qRT-PCR cycling conditions were as follows: 95 °C for 2 min, then 40 cycles, each of 10 s at 95 °C, 60 s at 56 °C, and 30 s at 70 °C. ∆ cycle threshold (*Ct*) was calculated by subtracting the Ct values of SNORD68 from the Ct values of the target miRs in all samples. Fold change was calculated using 2^−∆∆*Ct*^ for relative quantification.

### Routine lab biomarkers

Analysis: a complete blood count (CBC) was performed by a full automated hematology analyzer (Sysmex, KX21N, Kobe, Japan). Using a chemistry autoanalyzer device (Cobas Integra 400 Plus, Roche Diagnostics, Germany), following the manufacturer’s instructions, routine biochemical analysis of serum albumin, aspartate aminotransferase (AST), alanine aminotransferase (ALT), bilirubin (total and direct), alkaline phosphatase (ALP), gamma GT (GGT), total cholesterol (TC), triacylglycerol (TAG), and high-density lipoprotein-cholesterol (HDL-C) was performed. To measure plasma AFP, electro-chemiluminescence immunoassay (ECLIA) using a Cobas 6000, e601 module (Roche Diagnostics, Germany) was used. Finally, blood insulin was measured using the enzyme immunoassay (Hyperion Inc, Miami, FL).

### Inflammatory and resistance ratios (indices)

TAG/HDL-C ratio with a cutoff value of more than the apparently healthy control group is set diagnostic for insulin resistance (IR) (El-Mesallamy et al. 2011). IR is considered positive in obese, diabetic, and dyslipidemic patients, and having insulin levels of 18 mU/mL or more after glucose/meal (Kang et al. 2017).

### In silico database search and analysis

Exploring Research Resource Identifiers (RRIDs) and citing RRID portal, via *Tool*: RRID:SCR_003070 https://scicrunch.org/resources/data/source/nlx_144509-1/search.

### In silico identification of immune cells

To visualize closely related immune cells from release of the human peripheral blood mononuclear cell single cells or liver immune cells using Uniform Manifold Approximation and Projection (UMAP) (Becht et al. 2019). This was performed using the Human Universal Single Cell Hub (HUSCH) a scRNA-seq database http://husch.comp-genomics.org/#/info_tissue/ (accessed on Dec. 13th, 2022).

### Curated databases for prediction of monocytes surface antigens activation

Searched in relation to diseases pathogenesis and related metabolic and molecular pathways using the SIGnaling https://signor.uniroma2.it/ Network Open Resource (SIGNOR3.0) new release October 16th, 2022 (accessed on November 29th, 2022).

### Gene–gene interactions and pathways by bioinformatics analysis

#### Prediction of monocytes surface antigens

Monocytes surface antigens CD14 and CD16:FCGR3A top interacting genes to be predicted via gene interaction at University of California Santa Cruz (UCSC) (Fernandes et al. 2020). Genome Browser RRID: SCR_005780. Genomics institute http://genome.ucsc.edu/index.html (accessed on Dec. 13th, 2022).

### Statistical analysis

Data were tested for normality using Shapiro–Wilk online calculator [Internet]. Statistics Kingdom 2017 (accessed October, 2022). Available from https://www.statskingdom.com/shapiro-wilk-test-calculator.html (Date launched Nov. 2017, last update June 2022, and validated with R software). Normally distributed variables are presented as mean ± S.D and analyzed using two samples independent Students’ *t* test for comparison. For not-normally distributed variables, data are presented as median (inter-quartile range) as 1st–3rd quartiles: 25th–75th quartiles. Mann–Whitney (*U*) was conducted to compare between any two independent not-normally distributed groups. Qualitative data are presented as frequencies (*n*) and percentages (%). SPSS v17 (Chicago, IL, USA) software was used for analysis. Student’s *t* test and the Chi-square *χ*^2^ test were used to compare quantitative and qualitative normally distributed variables between the patients and control groups, respectively. Spearman’s rho correlation test was used to assess the association between quantitative non-parametric variables. Receiver operating characteristic (ROC) curve was performed to detect the best cutoff, sensitivities (SNs), specificities (SPs), with an area under the curve (AUC) calculated range from 0 to 1, where the higher the AUC, the better is the parameter in classifying the outcomes correctly. Logistic regression was performed to determine the independent association of the studied miRs and other parameters with LC progression to HCC. The significance level is set at value < 0.05 for *p* and the confidence level or confidence interval (CI) as 95% and 5%, respectively.

## Results

The study participants’ demographic and biochemical analysis data are shown in Table 1. The HCC group showed a significant increase in circulating intermediate monocytes when compared to LC group. In addition, the HCC group showed significant up-regulation of plasma hsa-miR-21-5p expression compared to LC group (median = 27.66-fold change vs 8.61-fold change from average expression, *p* < 0.001) and the control (*p* < 0.001). In addition, hsa-miR-155-5p expression was significantly higher in HCC patients in comparison to the cirrhotic patients (median = 3.18-fold change vs 1.81-fold change, *p* = 0.001) as well as to the control subjects (*p* = 0.001).

**Table 1:** Study participants’ demographic and clinical characteristics (unit) in liver cirrhosis group (*n* = 40) and HCC group (*n* = 39) compared to each other and to the apparently healthy control participants (*n* = 15)

Parameter (unit)	Groups, *n*			Significance		
	HCC, 39	LC, 40	Control, 15	*p*1	*p*2	*p3*
Gender M/F	27/12	28/12	11/4	NS	NS	NS
Age (years)	61.0 (56.0–67.0)	58.5 (54.25–65.0)	58.0 (55.0–60.0)	NS	NS	NS
BMI (kg/m^2^)	29.0 (27.0–31.0)	29.9 (27.55–33.2)	27.1 (26.7–27.8)	NS	0.008*	0.008*
D.M yes/no	15/24	16/24	0/15	NS	0.005*	< 0.001*
s. Insulin (mIU/L)	25.0 (15.7–42.5)	13.5 (5.98–20.37)	8.5 (5.9–10.7)	0.001*	< 0.001*	NS
s. Albumin (mg/dL)	3.4 (2.9–4.1)	2.7 (2.12–3.65)	3.3 (3.2–3.8)	0.009*	NS	0.035*
AST (U/L)	77.0 (62.0–105.0)	72.0 (62.0–78.0)	33.0 (30.0–43.0)	NS	< 0.001*	< 0.001*
ALT (U/L)	51.0 (42.0–65.0)	57.0 (50.0–63.7)	28.0 (24.0–38.0)	NS	< 0.001*	< 0.001*
Total bilirubin (mg/dL)	1.2 (0.9–2.0)	1.5 (1.0–3.07)	0.80 (0.6–1.0)	NS	< 0.001*	< 0.001*
Direct bilirubin (mg/dL)	0.70 (0.40–1.2)	0.8 (0.4–1.95)	0.30 (0.18–0.42)	NS	< 0.001*	< 0.001*
ALP (U/L)	110.0 (82.0–155.0)	120.0 (99.8–132.8)	46.0 (39.0–58.0)	NS	< 0.001*	< 0.001*
GGT (U/L)	60.0 (53.0–77.0)	67.0 (55.3–83.5)	19.0 (17.0–23.0)	NS	< 0.001*	< 0.001*
TC (mg/dL)	162.0 (122.0–220)	147.0 (112–181)	155.0 (152.0–162)	NS	NS	NS
TAG (mg/dL)	133.0 (94.0–193.0)	115.0 (76.8–147)	115.0 (99.0–123)	NS	NS	NS
HDL-C (mg/dl)	34.0 (26.0–40.0)	36.5 (30.5–41.75)	47.0 (43.0–51.0)	NS	< 0.001*	< 0.001*
TAG/HDL-C ratio	4.1 (2.6–6.7)	3.3 (2.34–4.59)	2.35 (2.2–2.7)	NS	< 0.001*	0.01*
NLR	2.5 (2.0–3.9)	2.1 (1.38–3.49)	0.93 (0. 33–1.42)	NS	< 0.001*	0.001*
PLR	111.4 (72.5–240.0)	74.5 (47.8–150.13)	128.4 (82.1–154.8)	NS	NS	NS
LMR	2.6 (1.4–4.25)	2.8 (1.60–3.92)	5.5 (4.1–6.0)	NS	0.001*	< 0.001*
AFP (ng/mL)	80 (13–305)	7.4 (4.5–10.37)	3.2 (2.7–6.8)	< 0.001*	< 0.001*	0.002*
Total monocytes %	6.7 (5.1–9.38)	6.0 (4.9–7.5)	3.0 (2.6–3.47)	NS	< 0.001*	< 0.001*
Classical monocytes %	4.5 (3.5–6.5)	4.0 (3.23–5.5)	1.9 (1.4–2.45)	NS	< 0.001*	< 0.001*
Intermediate monocytes %	1.16 (0.9–1.90)	0.6 (0.48–0.98)	0.15 (0.10–0.30)	< 0.001*	< 0.001*	< 0.001*
Non-classical monocytes %	0.56 (0.2–0.90)	0.5 (0.24–0.94)	0.24 (0.14–0.3)	NS	0.017*	0.010*
Hsa-miR-21-5p fold change	27.6 (6.9–69.5)	8.6 (3.9–11.3)	0.96 (0.94–1.0)	< 0.001*	< 0.001*	< 0.001*
Hsa-miR-155-5p fold change	3.1 (1.7–8.12)	1.8 (0.76–2.2)	1.07 (0.9–1.69)	0.001*	0.001*	NS

Data are median (inter-quartile range(1st–3rd quartile), statistics were computed using SPSS software, Mann–Whitney test was used (non-parametric data), *p1* for comparison between HCC and liver cirrhosis groups, *p2* for comparison between HCC and control, *p3* for comparison between liver cirrhosis and control

*ALT* alanine aminotransferase, *AST* aspartate aminotransferase, *AFP* alpha-fetoprotein, *BMI* body mass index, *HCC* hepatocellular carcinoma, *HDL* high-density lipoprotein, *GGT* gamma glutamyl transferase, *LC* liver cirrhosis, *PLR* platelet-to-lymphocyte ratio, *NLR* neutrophil-to-lymphocyte ratio, *LMR* lymphocyte-to-monocyte ratio, *TC* total cholesterol, *TAG* triacylglycerol

*Statistical significance *p* value < 0.05, NS, non-significant

The pathological characteristics of the HCC cases are shown in Table 2.

**Table 2:** Pathological characteristics of the studied HCC cases (*n* = 39) and LC cases (*n* = 40)

Pathology	Groups, *n* (%)		Statistics test, *p* value
	HCC, 39 (100%)	LC, 40 (100%)	
Ascites			*X*^2^ = 7.63, 0.05*
No	24 (61.5%)	16 (40.0%)	
Yes	15 (38.5%)	24 (60.0%)	
Liver size^a^	16.2(14–18)	12.6(10.47–14.27)	*U* test = 223.0, < 0.001*
Spleen size^a^	16.8(15.25–17.5)	15.5(13.42–20.95)	*U* test = 719, NS
Subclassification			
Liver disease Child score			*X*^2^ = 8.9, 0.012*
A = least severe	24 (61.5%)	12 (30.0%)	
B = moderately severe	10 (25.6%)	14 (35.0%)	
C = most severe	5 (12.8%)	14 (35.0%)	
BCLC classification			N.A
A = early stage	12 (30.8%)	–	
B = intermediate stage	10 (25.6%)	–	
C = advanced stage	12 (30.8%)	–	
D = terminal stage	5 (12.8%)	–	
Total	39 (100%)	–	

Data are number (%)

*NS* non-significant, *N.A* not applicable, *HCC* hepatocellular carcinoma, *LC* liver cirrhosis, *BCLC* Barcelona Clinic Liver Cancer

^a^Median (inter-quartile range (1st–3rd quartile)), statistics were computed using SPSS software

*Statistical significance *p*-value < 0.05

According to Child score data of all study patients (*n* = 79) presented in Table 3, hsa-miR-21-5p and hsa-miR-155-5p fold changes showed significant up-regulation in patients with early LC score A when compared to more advanced cases with Child score B and C. However, according to BCLC stage data in HCC patients (*n* = 39), no significant difference was detected between the HCC group with BCLC stage A and the more advanced BCLC stages concerning all study parameters (supplementary Table S1).

**Table 3:** Monocytes subsets frequencies, hsa-miR-21-5p and hsa-miR-155-5p fold changes in all study cases (*n* = 79) with liver cirrhosis background according to Child score

Group, *n*	LC with and without HCC, 79		*p* value
	Child score, *n*		
Parameter (unit)	A, 36	B and C, 43	
Total monocytes %	6.8(5.08–8.5)	6.0(5.0–8.1)	NS
Classical monocytes %	4.8(3.6–6.2)	3.9(3.3–5.9)	NS
Intermediate monocytes %	1.0(0.61–1.5)	0.90(0.60–1.3)	NS
Non-classical monocytes %	0.53(0.24–0.94)	0.50(0.21–0.77)	NS
Hsa-miR-21-5p fold change	20.3(9.9–39.5)	7.8(3.9–14.2)	0.002*
Hsa-miR-155-5p fold change	2.5(1.6–6.9)	1.8(0.76–2.6)	0.014*

Data are presented in median (inter-quartile range (1st–3rd quartile)), statistics were computed using SPSS software

*NS* non-significant, *HCC* hepatocellular carcinoma, *LC* liver cirrhosis

*Statistical significance *p* value < 0.05

Correlation studies between the investigated monocytes subsets with various biomarkers in all cases (*n* = 79) are presented in Table 4. Nonclassical monocytes frequency was negatively correlated with hsa-miR-155-5p (*r* = − 0.316, *p* = 0.005). The frequency of intermediate monocytes was positively correlated with hsa-miR-21-5p (*r* = 0.30, *p* = 0.007). A positive correlation was detected between intermediate monocytes frequency and insulin resistance (*r* = 0.266, *p* = 0.042), AFP (*r* = 0.258, *p* = 0.022), AST (*r* = 0.224, *p* = 0.047), and negative significant correlation with HDL (*r* = − 0.225, *p* = 0.046). Other non-significant correlations are presented in the supplementary Table S2.

**Table 4:** Spearman’s correlation coefficient among investigated frequency of monocytes subsets in all post-CHCV patients (*n* = 79)

Monocytes percentage	Post-CHCV G4 patients (*n* = 79)							
	Total		Classical		Intermediate		Non-classical	
Parameter	*r*	*p* value	*r*	*p* value	*R*	*p* value	*R*	*p* value
Insulin resistance	0.015	NS	− 0.012	NS	0.266	0.042*	0.002	NS
AFP (ng/mL)	0.106	NS	0.078	NS	0.258	0.022*	0.044	NS
AST (U/L)	0.029	NS	− 0.075	NS	0.224	0.047*	0.081	NS
HDL-C (mg/dL)	0.059	NS	0.000	NS	0.225	0.046*	0.191	NS
Hsa-miR-21-5p	0.066	NS	0.051	NS	0.300	0.007*	0.195	NS
Hsa-miR-155-5p	0.093	NS	0.013	NS	0.181	NS	− 0.316	0.005*

Spearman correlation coefficient (*r*) was calculated using SPSS software

*NS* non-significant, *AST* aspartate aminotransferase, *AFP* alpha-feto protein, *HDL* high-density lipoprotein

*Significant correlation at *p* < 0.05 level (2-tailed)

Correlation studies between the inflammatory indices with various biomarkers in all cases (*n* = 79) are presented in the supplementary Table S3, where significant negative correlation was shown between absolute monocytic count (AMC) and hsa-miR-155-5p fold changes (*r* = − 0.233, *p* = 0.039).

**Fig. 2:**
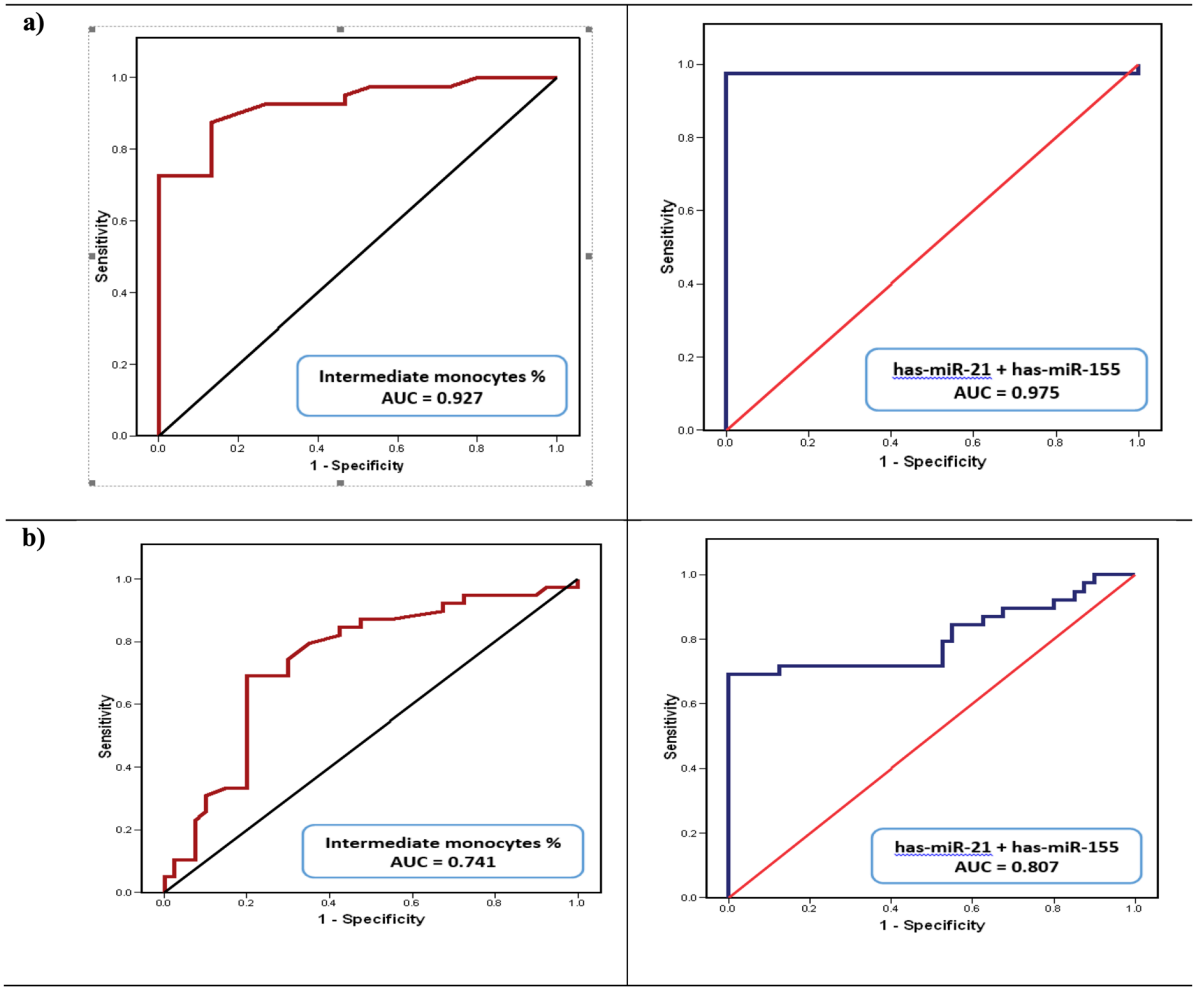
ROC curve for the discriminative ability of the investigated miRs expression level combined (right) hsa-miR-21-5p + hsa-miR-155-5p or the intermediate monocytes subsets % (left) **a** to detect liver cirrhosis development and **b** to detect liver cirrhosis cases from HCC

The discriminative ability of the studied parameters to differentiate LC cases from healthy control and to differentiate HCC cases from LC cases, calculated from the ROC curve. As depicted in Table 5 and Fig. 2, according to the ROC curves, the discriminative power to differentiate HCC from LC cases after combining hsa-miR-21-5p and hsa-miR-155-5p yielded SN = 84.6%, SP = 45%, and AUC = 0.80.

**Table 5:** The discriminative ability of the studied markers to differentiate liver cirrhosis cases from healthy controls and HCC from liver cirrhosis cases

Variable	%				*p* value
	Cutoff	AUC	SN	SP	
Discriminative ability to differentiate LC cases from healthy controls					
Intermediate monocytes percentage	> 0.38	0.927	82.5	86.7	< 0.001*
Hsa-miR-21-5p fold change	> 1.55	0.977	97.5	100	< 0.001*
Hsa-miR-155-5p fold change	> 1.17	0.618	67.5	60	NS
Hsa-miR-21-5p + hsa-miR-155-5p	–	0.975	97.5	100	< 0.001*
Intermediate monocytes percentage + hsa-miR-21-5p	–	1.00	100	100	< 0.001*
Intermediate monocytes percentage + hsa-miR-155-5p	–	0.935	90	86.7	< 0.001*
AFP (ng/mL)	> 5.9	0.777	62.5	73.3	0.002*
NLR	> 1.14	0.805	80.0	73.3	0.001*
LMR	< 5.09	0.813	90.0	66.7	< 0.001*
Discriminative ability to differentiate HCC cases from LC					
Intermediate monocytes percentage	0.65	0.741	87.2	52.5	< 0.001*
Hsa-miR-21-5p fold change	> 7.3	0.8	74	45	< 0.001*
Hsa-miR-155-5p fold change	> 1.8	0.7	72	48	< 0.01*
Hsa-miR-21-5p + hsa-miR-155-5p	–	0.807	84.6	45	< 0.001*
Intermediate monocytes percentage + hsa-miR-21-5p	–	0.844	79.5	75	< 0.001*
Intermediate monocytes percentage + hsa-miR-155-5p	–	0.766	76.9	75	< 0.001*
AFP (ng/mL)	> 23.3	0.85	69	100	< 0.001*

*AFP* alpha-feto protein, *AUC* area under the curve, *SN* sensitivity, *SP* specificity

*Significance at *p* < 0.05 level (2-tailed)

While a better specificity was achieved after combining hsa-miR-155-5p and frequency of intermediate monocytes which yielded SN = 76.9%, SP = 75%, and AUC = 0.766. Moreover, combining hsa-miR-21-5p and the frequency of intermediate monocytes yielded SN = 79.5%, SP = 75%, and AUC = 0.844. In comparison, AFP yielded a lower SN = 69% and 100% SP with AUC = 0.85.

Logistic regression analysis as depicted in Table 6 proved that the circulating classical, intermediate monocytes frequencies and hsa-miR-21-5p were independent risk factors for LC progression to HCC after adjustment for confounders (age, BMI, RBS, AFP, and lipids).

**Table 6:** Logistic regression analysis using monocytes subsets percentage, hsa-miR-21-5p and hsa-miR-155-5p expression level-fold change, and the inflammatory ratios as predictors of liver cirrhosis progression to HCC (*n* = 79) after adjustment for the confounders (AFP, age, BMI, lipids, and RBS)

	*p* value	OR	95% CI	
			Lower	Upper
Classical monocytes percentage	0.026*	4.310	1.192	15.585
Intermediate monocytes percentage	0.045*	6.721	1.047	43.146
Non-classical monocytes percentage	NS	1.509	0.736	3.093
Hsa-miR-21-5p fold change	0.001*	1.183	1.069	1.309
Hsa-miR-155-5p fold change	NS	0.990	0.952	1.030
AFP (ng/mL)	0.016*	1.182	1.032	1.353
Age (years)	0.029*	1.164	1.015	1.335
BMI (kg/m^2^)	0.050*	0.806	0.649	1.000
TAG (mg%)	NS	1.004	0.982	1.025
TC (mg%)	NS	1.000	0.981	1.021
RBS (mg%)	NS	0.988	0.974	1.003

*BMI* body mass index, *CI* confidence interval, *HCC* hepatocellular carcinoma, *HDL* high-density lipoprotein, *OR* odds ratio, *RBS* random blood sugar, *TAG* triacylglycerol, *TC* total cholesterol

*Significant *p* value < 0.05

### In silico database analyses for identification of immune cells from blood and liver

**Fig. 3:**
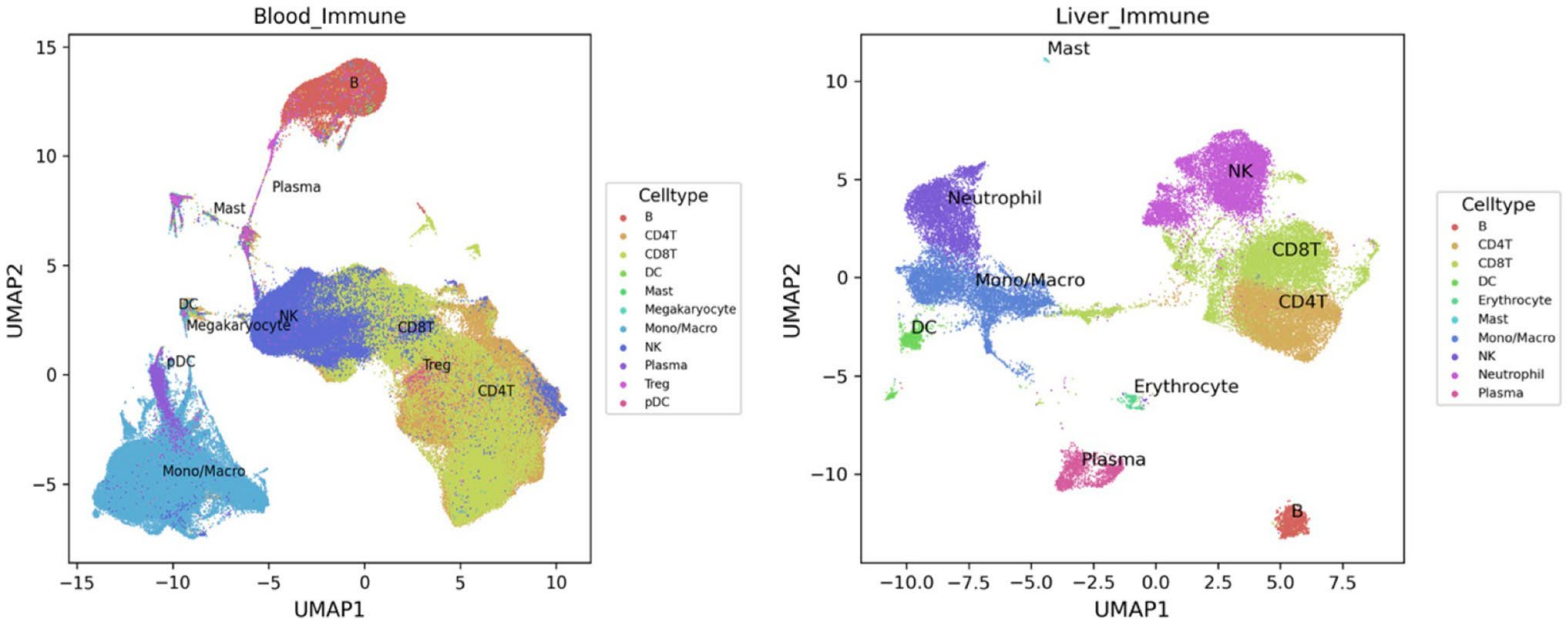
Immune cell types expression analysis by the Human Universal Single Cell Hub (HUSCH) from blood and liver http://husch.comp-genomics.org/#/info_tissue/Blood and http://husch.comp-genomics.org/#/info_tissue/Liver, respectively (accessed on Dec. 13th, 2022). *UMAP*, Uniform Manifold Approximation and Projection

Figure 3 illustrates blood circulating and liver immune cells pattern of clustering.

Blood circulating immune cells annotation details Dataset: 21, CellNumber: 483286, Celltype: B, *CD4*^+^ T cells, *CD8*^+^ T cells dendritic cells (DC), Mast, Megakaryocyte, *CD14*^+^ monocytes, *FCGR3A*^+^ monocytes, Myofibroblast, Neutrophil, NK, Plasma, and T-regulatory (Treg) cells (http://husch.comp-genomics.org/#/info_tissue/Blood).

Liver immune cells annotation details Dataset: 7, CellNumber: 59993, Celltype: B, CD4T, CD8T, Cholangiocyte, DC, Endothelial, Epithelial, Erythrocyte, Hepatic Oval, Hepatocyte, Kupffer, Mast, Mesenchymal, Mono/Macro, Muscle, Neutrophil, NK, Plasma, Portal Endothelial, Smooth Muscle, and Treg (http://husch.comp-genomics.org/#/info_tissue/Liver) studied by the Human Universal Single Cell Hub (HUSCH) (accessed on Dec. 13th, 2022). [UMAP, Uniform Manifold Approximation and Projection.]

The database analysis clarifies that abnormality of monocyte in the peripheral blood will be reflected on the liver and will play a part in the pathogenesis of liver inflammation due to confirmed dynamic circulation of monocytes between the peripheral blood and the liver (Melino et al. 2016; Gadd et al. 2016).

### Monocyte activation pathway bioinformatics

SIGNOR3.0 searched in relation to diseases pathogenesis pathways (accessed on November 29^th^, 2022), where the transcription activator regulator SPI1 (involved in blood cells differentiation and activation) up-regulates the monocyte differentiation antigen CD14 expression via transcriptional regulation https://signor.uniroma2.it/relation_result.php?id=P08571. However, hsa-miR-155 down-regulates SPI1 via post-transcriptional repression https://signor.uniroma2.it/relation_result.php?id=P17947&organism=human.

### Monocytes surface antigens gene–gene interactions and pathways from curated databases and text-mining (Fig. 4)

**Fig. 4:**
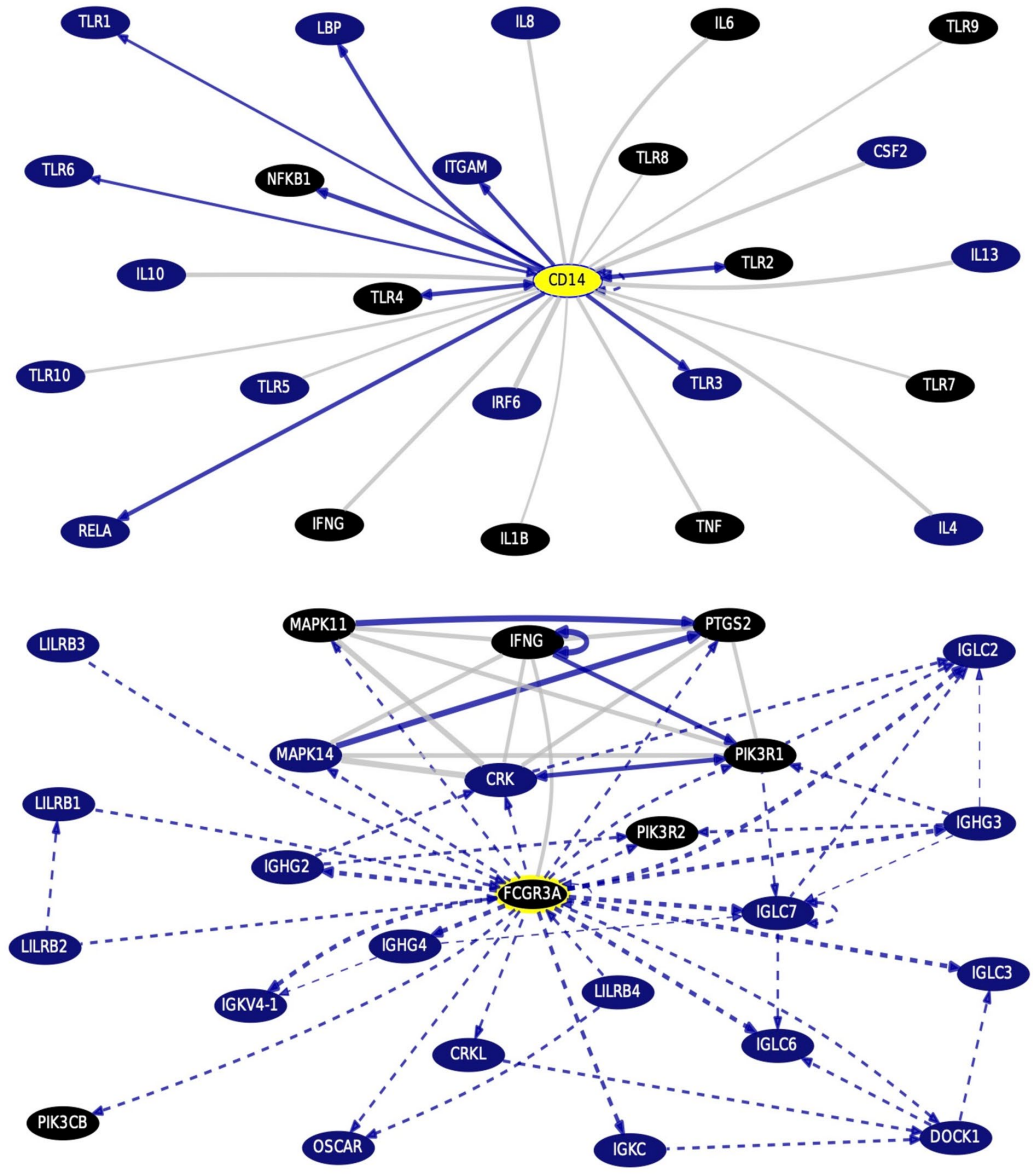
Monocytes surface antigens CD14 and CD16A:FCGR3A top interacting genes, with highlighting the drug bank interaction, obtained via gene interactions and pathways from curated databases and text-mining using UCSC Genome Browser Gene Interaction Graph (accessed on November 29th, 2022) http://genome.ucsc.edu/cgi-bin/hgGeneGraph?gene=CD14&1=OK&supportLevel=text&geneCount=25&geneCount=25&geneAnnot=drugbank&1=OK&lastGene=MIR21 and http://genome.ucsc.edu/cgi-bin/hgGeneGraph?supportLevel=text&geneCount=25&geneAnnot=drugbank&1=OK&lastGene=MIR21&gene=FCGR3A, respectively. [Black colored genes, being treatment hits by DrugBank]

Via gene interaction on UCSC genomics institute http://genome.ucsc.edu/cgi-bin/hgGeneGraph?gene=CD14&1=OK&supportLevel=text&geneCount=25&geneCount=25&geneAnnot=drugbank&1=OK&lastGene=MIR21 and http://genome.ucsc.edu/cgi-bin/hgGeneGraph?supportLevel=text&geneCount=25&geneAnnot=drugbank&1=OK&lastGene=MIR21&gene=FCGR3A for monocytes surface antigens CD14 and CD16A:FCGR3A, respectively.

The monocytes surface antigen CD14 top interacting genes are TLRs, cytokines IL-6, IL-1Beta, TNF, interferon-gamma, and the NF-_*K*_B, targeted by anti-TNF drugs and anti-cytokines therapy (DrugBank). However, the surface antigen CD16A:FCGR3A top interacting genes are interferon-gamma, mitogen-activated protein 3 kinase 11 (MAP3K11), PIK3R1 and R2, targeted by caffeine, isoprenaline, and glucosamine (DrugBank).

## Discussion

In the context of immune perturbation that drives LC progression to HCC, the relationship between the pro-inflammatory plasma molecular biomarkers, hsa-miR-21-5p and hsa-miR-155-5p and the plasticity of circulating monocytes is still not well understood. Therefore, our goal was to find answers in this field as a step towards ncRNA precision medicine.

Current study revealed significant increase in the frequency of intermediate monocytes in the peripheral blood in HCC group when compared to LC group, which points out the role of this pro-inflammatory subpopulation in LC progression to HCC. According to Melino et al. (2016), monocytes will dynamically move to and from the liver sharing in pathogenesis of liver disease. Increased hepatic monocyte recruitment and systemic activation states may be influenced by injury-induced signals (Gadd et al. 2016). There was no significant difference between LC and HCC groups regarding non-classical monocytes. However, there was a significant reduction in the non-classical monocyte subsets frequency in LC and HCC groups when compared to normal control group. Significant reduction in the non-classical monocyte subsets frequency leads to lack of their anti-tumoral and anti-inflammatory impact as proposed by Ali et al. (2022), and therefore, facilitates LC and HCC development. The current study results showed no discernible difference between the HCC and LC groups, in terms of the frequency of circulating total, classical, and non-classical monocytes. Blood inflammation indices are cost-effective and easily available, but, unfortunately, non-specific to tumor (Zhu and Zhou 2022).

In the current study, significant differences were seen in neutrophil-to-lymphocyte ratio (NLR) and lymphocyte-to-monocyte ratio (LMR) between LC patients and controls as well as between HCC patients and controls, while no differences were detected between LC and HCC groups. Zhu and Zhou (2022) documented NLR ability to distinguish LC from healthy controls but could not discriminate cirrhotic HCC from LC. In addition, LMR was suggested to be useful to determine the outcome of cirrhotic patients by Hsu et al. (2021). In contrast, Du et al. (2018) found that elevated NLR is associated with HCC development in cirrhotic patients with HBV who underwent splenectomy for hypersplenism. Yilma et al. (2022) suggested that NLR was a component of HCC development and recurrence risk models in the context of CHCV. Currently, PLR showed no significant differences among the three groups, which disagrees with Catanzaro et al. (2020) who inferred PLR to be used as a predictive marker for LC.

Our correlation data results revealed a positive correlation between the frequency of intermediate monocytes percentage and insulin resistance (IR). Keeping in mind that the IR is a state of chronic low-level inflammation (Scarpellini and Tack 2012) and intermediate-monocyte population has already been related to some inflammatory disease (Wong et al. 2011), via higher prevalence of the monocyte surface activation inflammatory marker CD14 (Shikuma et al. 2014). Accordingly, it has been shown that experimental inhibition of monocyte recruitment to the liver through blocking the C–C motif chemokine receptor 2 (CCR2), ameliorates both the IR and hepatic inflammation (Krenkel et al. 2018).

In addition, intermediate monocytes percentage showed a significant correlation with AFP (*r* = 0.258, *p* = 0.022). Our results agree with Kong et al. (2012) that AFP level is associated with monocyte activation and its phagocytosis ability. On the other hand, it was claimed that the immunomodulating properties of tumor-derived AFP (tAFP) could induce immune-escape through inhibiting monocyte-derived dendritic cells (DC) function (Wang and Wang 2018). Interestingly, Munson et al. (2022) observed the in vitro tAFP ability to suppress monocyte function, rather than frequency, via suppressing their ability to produce TNFα and IL-1β.

Intermediate monocytes percentage showed a significant negative correlation with HDL-C (*r* = − 0.225, *p* = 0.046) agreeing with Rogacev et al. (2014). Idzkowska et al. (2015) provided evidence that inflammation could induce lipid dysregulation mainly through the modulation of monocytes recruitment and activation. Monocytes priming was demonstrated in dyslipidemic LC or HCC patients (Patel et al. 2017). Therefore, monocytes may present one important component, via atherosclerosis development, during the liver fibrotic stage preceding or early during LC. According to Martín-Sierra et al. (2020), intermediate-monocyte subsets through their pro-inflammatory role are related to HCC tumorigenicity in CHCV G4 Egyptian patients. Since hsa-miR-21-5p over-expression, in the disease groups, was positively correlated with intermediate monocytes percentage, therefore, we can hypothesize that up-regulated hsa-miR-21-5p may be involved in monocytes differentiation (Cekaite et al. 2010). It is noteworthy to mention, that we have studied adipokines single nucleotide polymorphisms (SNPs), several apoptosis/autophagy genes and their role on IR or inflammation, immunity, and carcinogenesis or the reverse, as well as several Ils or vitamin D SNPs influence on various types of cancers, common in Egypt, as an attempt of study “Cancer Genetics in The Egyptian Population” (El-Mesallamy et al. 2012; El-Derany et al. 2016; El Mesallamy et al. 2014; Ali et al. 2021; Youssef and Hamdy 2017). However, nowadays, we are into epigenetics.

Correlation results obtained in the study between hsa-miR-21-5p and/or hsa-miR-155-5p and the monocyte subsets, suggest a coincidence and/or an interaction and provide evidence of the inflammatory roles of the studied miRs. These findings agree with Mahesh and Biswas (2019) study on hsa-miR-155-5p and Madhyastha et al. (2021) study on hsa-miR-21-5p. According to the Mattoscio et al. (2021), non-classical monocytes exert anti-tumoral properties manifested as cytotoxicity, preventing metastasis, autophagy, NK cells recruitment, and Treg suppression. However, significant reduction in the non-classical monocyte subsets percentage will be manifested as lack of their anti-tumoral impact, therefore, unfortunately, HCC development commences.

Regarding ROC curves analysis, hsa-miR-21-5p was shown to be superior to hsa-miR-155-5p as a plasma molecular marker for identifying LC cases from healthy cases and combining both hsa-miR-21-5p + hsa-miR-155-5p provided an accepted discriminative sensitivity for HCC cases identification from LC. The low specificity associating hsa-miR-21-5p and hsa-miR-155-5p as diagnostic markers is explained by their nature as pro-inflammatory markers associating variety of pathological conditions (Mahesh and Biswas 2019) Nerveless, it was observed that combining the frequency of intermediate monocytes increases their specificity as diagnostic markers to HCC linked to post-CHCV-LC. Moreover, logistic regression analysis proved that circulating classical and intermediate monocytes percentage and hsa-miR-21-5p were independent predictors of HCC progression from cirrhotic background after adjustment for the confounders (age, BMI, RBS, AFP, and lipids). This confirms that peripheral blood monocytes play a role in the pathogenesis of liver inflammation due to confirmed dynamic circulation of monocytes between the peripheral blood and the liver. Circulating monocytes constantly move to and from the liver but are recruited in greater numbers when there is liver inflammation and this process may be influenced by injury-induced signals (Melino et al. 2016 and Gadd et al. 2016).

Monocyte activation pathway informatics (SIGNOR3.0) revealed that the transcription factor SPI1 could up-regulate CD14 expression on monocytes surface. Hsa-miR-155 could post-transcriptionally down-regulate SPI. This may explain our finding of hsa-miR-155-5p negative correlation to non-classical monocytes subtypes characterized with dim CD14 expression, which could be claimed from one side to the indirect suppressive effect of the up-regulated hsa-miR-155-5p on monocytes surface-expressed CD14, mediated via SPI1. Kyoto Encyclopedia of Genes and Genomes KEGG targeted pathways search for *MiR21* and *MIR155* genes Clusters/Heatmap using DIANA TOOLS Mirpath reverse search and genes that share domains determined via GenesLikeMe. *MiR21* and *MIR155* genes are related to each other. *MIR155* gene is related to and is targeted with genes involved in inflammation *NF-kB, STAT3, IL-6, TNF, MIR21, MAPK8*, and *TLR4*.

*Limitation* The current study did not include the predictive survival role of the investigated miRs panel; hsa-miR-21-5p and hsa-miR-155-5p in the CHCV G4-linked to HCC patients’ cohort (a prospective study is prepared by our group, currently).

*Strength(s)-related to the current research* Up to our knowledge, this study is the first to describe the diagnostic utility of hsa-miR-21-5p or hsa-miR-155-5p and as panel, in combination with AFP, for an enhanced and, hopefully, early diagnosis of clinical CHCV G4-related HCC and LC. Hsa-miR-21-5p/hsa-miR-199a-5p ratios are proved, clinically, in the current study, as diagnostic for AFP-negative HCC cases (Eldosoky et al. 2023).

*Recommendations* Hsa-miR-21-5p and/or hsa-miR-155-5p is linked to altered monocytes distribution on top of LC and HCC, for finding potential therapeutic option(s), based on the immune cells’ perturbation in blood. Considering hsa-miR-21-5p and/or hsa-miR-155-5p as potential precision nc-epigenetic therapeutic target(s) for CHCV-G4-related HCC and/or LC treatment, based on blood monocytes sub-classification, after proving the suggested mechanism experimentally.

*Sustainability plan* Blocking hsa-miR-21-5p and/or hsa-miR-155-5p target genes obtained from gene–gene interaction network algorithms and KEGG pathways in silico curated databases. These genes if being targeted will present a promising future potential treatment option(s) and treatment-based on ncRNA, a step toward precision health.

## Conclusion

Monocyte subsets differentiation in HCC was linked to hsa-miR-21-5p and hsa-miR-155-5p. Combined up-regulation of intermediate monocytes frequency and hsa-miR-21-5p expression could be considered a sensitive indicator of post-CHCV-LC progression to HCC. Combined up-regulation of hsa-miR-21-5p + hsa-miR-155-5p can serve as a molecular biomarker for diagnosis of HCC linked to post-CHCV-LC with accepted sensitivity. Altered intermediate monocytes frequency is linked to deregulated lipid metabolism and insulin resistance in LC patients. Circulating classical monocytes, intermediate monocytes frequencies and hsa-miR-21-5p were shown to be independent risk factors for HCC evolution post-CHCV-LC. Drugs for *MIR21* and *MIR155* genes from GeneCards, DrugBank, PharmGKB, DGIdb, IUPHAR, and Novoseek are cisplatin and Cobomarsen, respectively. Drugs targeting *MiR21* and *MIR155* down-stream related/interacting genes and the monocytes surface antigen *CD14* gene are lovastatin or anti-inflammatory, anti-cytokine; anti-TNF-alpha, caffeine, and glucosamine.

## Supplementary Information

Below is the link to the electronic supplementary material.

Supplementary file1 (DOCX 31 KB)

## Data Availability

The original contributions presented in the study are included in the manuscript. Further inquiries can be directed to the corresponding author.

## Abbreviations

AFP: Alpha-fetoprotein
ALP: Alkaline phosphatase
ALT: Alanine aminotransferase
AMC: Absolute monocytic count
AST: Aspartate aminotransferase
AUC: Area under the curve
BCL2: B cell lymphoma 2
BCLC: Barcelona clinic liver cancer
BMI: Body mass index
C.I: Confidence level or interval
CAID: Cirrhosis-associated immune dysfunction
CBC: Complete blood count
CCR2: Chemokine receptor C–C motif chemokine receptor 2
Ct: Cycle threshold
CL2: Chemokine (C–C motif) ligand 2
CD: Cluster of differentiation
cDNA: Complementary DNA
CHCV: Chronic hepatitis C virus
CT: Computed tomography
CYR61: Cysteine-rich angiogenic inducer 61
DAMPs: Damage-associated molecular patterns
DN: Dendritic cells
D.M: Diabetes mellitus
ECLIA: Electro-chemiluminescence immunoassay
EDTA: Ethylenediaminetetraacetic acid
EGF: Epidermal growth factor
ELISA: Enzyme-linked immunosorbent test
FC: Flow cytometry
FCGR3A: Flow cytometry gamma receptor IIIa
FS: Forward scatter
G4: Genotype 4
GGT: Gamma glutamyl transferase
HBV: Hepatitis B virus
HCC: Hepatocellular carcinoma
HCV: Hepatitis C virus
HDL: High-density lipoprotein
HIF-1: Hypoxia-induced factor-1
Hsa: Homo sapiens
HUSCH: Human universal single-cell hub
I.C: Informed consent
IGF1: Insulin-like growth factor 1
IL-6: Interleukin-6
INR: International normalized ratio
INS: Insulin
IR: Insulin resistance
KEGG: Kyoto Encyclopedia of Genes and Genomes
LC: Liver cirrhosis
LMR: Lymphocyte-to-monocyte ratio
LN: Lymph node
MAPK8: Mitogen-activated protein kinase 8
MAP3K11: Mitogen-activated protein 3 kinase 11
miRs: MicroRNAs
nc: Non-protein coding
NF-_*k*_B: Nuclear factor kappa-B cell
NK: Natural killer
NLR: Neutrophil-to-lymphocyte ratio
OR: Odds ratio
PI3K: Phosphatidyl inositol 3 kinases
PLR: Platelets-to-lymphocytes ratio
PV: Portal vein
qRT-PCR: Quantitative real-time PCR
RBS: Random blood sugar
REC: Research Ethics Committee
ROC: Receiver operating characteristic
SMAD2: Suppressor of mothers against decapentaplegic 2
SN: Sensitivities
SNORD68: Small nucleolar RNA, C/D box 68
RRIDs: Research resource identifiers
SNPs: Single-nucleotide polymorphisms
SP: Specificities
SPSS: Statistical package for social science software
STAT3: Signal transducer and activator of transcription 3
TAG: Triacylglycerol
tAFP: Tumor-derived AFP
TC: Total cholesterol
TLR4: Toll-like receptor 4
TP: Tumor protein
Treg: T-regulatory
UCSC: University of California Santa Cruz
UMAP: Uniform Manifold Approximation and Projection

## References

[CR1] Albillos A, Martin-Mateos R, Van der Merwe S et al (2022) Cirrhosis-associated immune dysfunction. Nat Rev Gastroenterol Hepatol 19(2):112–134. 10.1038/s41575-021-00520-7 34703031 10.1038/s41575-021-00520-7

[CR2] Ali NA, Hamdy NM, Gibriel AA et al (2021) Investigation of the relationship between CTLA4 and the tumor suppressor RASSF1A and the possible mediating role of STAT4 in a cohort of Egyptian patients infected with hepatitis C virus with and without hepatocellular carcinoma. Arch Virol 166:1643–1651. 10.1007/s00705-021-04981-8 33796885 10.1007/s00705-021-04981-8

[CR3] Ali F, Hammad R, Kotb FM et al (2022) Flow cytometry assessment of monocyte subsets alteration in hepatocellular carcinoma post hepatitis C virus infection. Egypt J Immunol 29(4):33–45. 10.55133/eji.290404 36197152

[CR4] Becht E, McInnes L, Healy J et al (2019) Dimensionality reduction for visualizing single-cell data using UMAP. Nat Biotechnol 37:38–44. 10.1038/nbt.4314 10.1038/nbt.4314 30531897

[CR5] Catanzaro R, Sciuto M, Lanzafame C et al (2020) Platelet to lymphocyte ratio as a predictive biomarker of liver fibrosis (on elastography) in patients with hepatitis C virus (HCV)-related liver disease. Indian J Gastroenterol 39(3):253–260. 10.1007/s12664-020-01038-7 32833144 10.1007/s12664-020-01038-7

[CR6] Caviglia GP, Ciruolo M, Abate ML et al (2020) Alpha-fetoprotein, protein induced by vitamin K absence or antagonist II and glypican-3 for the detection and prediction of hepatocellular carcinoma in patients with cirrhosis of viral etiology. Cancers 12(11):3218. 10.3390/cancers12113218 33142893 10.3390/cancers12113218 PMC7692611

[CR7] Cekaite L, Clancy T, Sioud M (2010) Increased miR-21 expression during human monocyte differentiation into DCs. Front Biosci Elite 2(3):818–828. 10.2741/e143 10.2741/e143 20515755

[CR8] China L, Maini A, Skene SS et al (2018) Albumin counteracts immune-suppressive effects of lipid mediators in patients with advanced liver disease. Clin Gastroenterol Hepatol 16(5):738-747.e7. 10.1016/j.cgh.2017.08.027 28859868 10.1016/j.cgh.2017.08.027 PMC6168974

[CR9] Coillard A, Segura E (2019) In vivo differentiation of human monocytes. Front Immunol 10:1–7. 10.3389/fimmu.2019.01907 31456804 10.3389/fimmu.2019.01907 PMC6700358

[CR10] Demerdash HM, Hussien HM, Hassouna E, Arida EA (2017) Detection of microRNA in hepatic cirrhosis and hepatocellular carcinoma in hepatitis C genotype-4 in Egyptian patients. Biomed Res Int 2017:1806069. 10.1155/2017/1806069 28642869 10.1155/2017/1806069 PMC5469990

[CR11] Dogra P, Rancan C, Ma W et al (2020) Tissue determinants of human NK cell development, function, and residence. Cell 180(4):749–763. 10.1016/j.cell.2020.01.022 32059780 10.1016/j.cell.2020.01.022 PMC7194029

[CR12] Du Z, Dong J, Bi J et al (2018) Predictive value of the preoperative neutrophil-to-lymphocyte ratio for the development of hepatocellular carcinoma in HBV-associated cirrhotic patients after splenectomy. PLoS ONE 13(4):e0195336. 10.1371/journal.pone.0195336. (**Erratum in: PLoS One 201914(4): e0215183**) 29621282 10.1371/journal.pone.0195336 PMC5886527

[CR13] El Mesallamy HO, Rashed WM, Hamdy NM et al (2014) High-dose methotrexate in Egyptian pediatric acute lymphoblastic leukemia: the impact of ABCG2 C421A genetic polymorphism on plasma levels, what is next? J Cancer Res Clin Oncol 140:1359–1365. 10.1007/s00432-014-1670-y 24718721 10.1007/s00432-014-1670-y PMC11823488

[CR14] El-Derany MO, Hamdy NM, Al-Ansari NL, El-Mesallamy HO (2016) Integrative role of vitamin D related and Interleukin-28B genes polymorphism in predicting treatment outcomes of chronic Hepatitis C. BMC Gastroenterol 16:19. 10.1186/s12876-016-0440-5 26911666 10.1186/s12876-016-0440-5 PMC4765184

[CR15] Eldosoky MA, Hammad R, Elmadbouly AA, Aglan RB, AbdelHamid SG, Alboraie M et al (2023) Diagnostic significance of hsa-miR-21-5p, hsa-miR-192-5p, hsa-miR-155–5p, hsa-miR-199a-5p panel and ratios in hepatocellular carcinoma on top of liver cirrhosis in HCV infected patients. Int J Mol Sci 24:3157. 10.3390/ijms24043157 36834570 10.3390/ijms24043157 PMC9962339

[CR16] El-Hefny M, Fouad S, Hussein T et al (2019) Circulating microRNAs as predictive biomarkers for liver disease progression of chronic hepatitis C (genotype 4) Egyptian patients. J Med Virol 91(1):93–101. 10.1002/jmv.25294 30133717 10.1002/jmv.25294

[CR17] El-Mesallamy HO, Hamdy NM, Rizk HH, El-Zayadi A-R (2011) Apelin serum level in egyptian patients with chronic hepatitis C. Mediat Inflamm 2011:70303. 10.1155/2011/703031 10.1155/2011/703031 PMC3189591 22007137

[CR18] El-Mesallamy HO, Hamdy NM, Zaghloul AS, Sallam MA-A (2012) Serum retinol binding protein-4 and neutrophil gelatinase-associated lipocalin are interrelated in pancreatic cancer patients. Scand J Clin Lab Investig 72(8):602–607. 10.3109/00365513.2012.723135 23020231 10.3109/00365513.2012.723135

[CR19] Faure-Dupuy S, Lucifora J, Durantel D (2017) Interplay between the hepatitis B virus and innate immunity: from an understanding to the development of therapeutic concepts. Viruses 9(5):95. 10.3390/v9050095 28452930 10.3390/v9050095 PMC5454408

[CR20] Fernandes JD, Hinrichs AS, Clawson H et al (2020) The UCSC SARS-CoV-2 genome browser. Nat Genet 52(10):991–998. 10.3390/v9050095 32908258 10.1038/s41588-020-0700-8 PMC8016453

[CR21] Gadd VL, Patel PJ, Jose S et al (2016) Altered peripheral blood monocyte phenotype and function in chronic liver disease: implications for hepatic recruitment and systemic inflammation. PLoS ONE 11(6):e0157771. 10.1371/journal.pone.0157771 27309850 10.1371/journal.pone.0157771 PMC4911107

[CR22] Georg P, Astaburuaga-García R, Bonaguro L et al (2022) Complement activation induces excessive T cell cytotoxicity in severe COVID-19. Cell 185(3):493–512. 10.1016/j.cell.2021.12.040 35032429 10.1016/j.cell.2021.12.040 PMC8712270

[CR23] Gómez-olarte S, Bolaños NI, Echeverry M et al (2019) Intermediate monocytes and cytokine production associated with severe forms of Chagas disease. Front Immunol 10:1–10. 10.3389/fimmu.2019.01671 31379862 10.3389/fimmu.2019.01671 PMC6658923

[CR24] Gu Y, Bi Y, Wei H et al (2021) Expression and clinical significance of inhibitory receptor Leukocyte-associated immunoglobulin-like receptor-1 on peripheral blood T cells of chronic hepatitis B patients: a cross-sectional study. Medicine 100:e26667. 10.1097/md.0000000000026667 34398030 10.1097/MD.0000000000026667 PMC8294879

[CR25] Guo P, Qiao F, Huang D et al (2020) MiR-155–5p plays as a “janus” in the expression of inflammatory cytokines induced by T-2 toxin. Food Chem Toxicol 140:111258. 10.1016/j.fct.2020.111258 32240701 10.1016/j.fct.2020.111258

[CR26] Gupta M, Akhtar J, Sarwat M (2022) MicroRNAs: regulators of immunological reactions in hepatocellular carcinoma. Semin Cell Dev Biol 124:127–133. 10.1016/j.semcdb.2021.05.025 34049801 10.1016/j.semcdb.2021.05.025

[CR27] Hammad R, Aglan RB, Mohammed SA et al (2022) Cytotoxic T cell expression of leukocyte-associated immunoglobulin-like receptor-1 (LAIR-1) in viral hepatitis C-mediated hepatocellular carcinoma. Int J Mol Sci 23(20):12541. 10.3390/ijms232012541 36293412 10.3390/ijms232012541 PMC9604124

[CR28] Hsu YC, Yang YY, Tsai IT (2021) Lymphocyte-to-monocyte ratio predicts mortality in cirrhotic patients with septic shock. Am J Emerg Med 40:70–76. 10.1016/j.ajem.2020.11.071 33348227 10.1016/j.ajem.2020.11.071

[CR29] Idzkowska E, Eljaszewicz A, Miklasz P et al (2015) The role of different monocyte subsets in the pathogenesis of atherosclerosis and acute coronary syndromes. Scand J Immunol 82(3):163–173. 10.1111/sji.12314 25997925 10.1111/sji.12314

[CR30] Irvine KM, Ratnasekera I, Powell EE, Hume DA (2019) Causes and consequences of innate immune dysfunction in cirrhosis. Front Immunol 10:293. 10.3389/fimmu.2019.00293. (**Erratum in: Front Immunol 10:818**) 30873165 10.3389/fimmu.2019.00293 PMC6401613

[CR31] Joharji H, Alkortas D, Ajlan A et al (2022) Efficacy of generic sofosbuvir with daclatasvir compared to sofosbuvir/ledipasvir in genotype 4 hepatitis C virus: a prospective comparison with historical control. Health Sci Rep 6(1):e980. 10.1002/hsr2.980 36514327 10.1002/hsr2.980 PMC9731361

[CR32] Kang B, Yang Y, Lee EY et al (2017) Triglycerides/glucose index is a useful surrogate marker of insulin resistance among adolescents. Int J Obes 41:789–792. 10.1038/ijo.2017.14 10.1038/ijo.2017.14 28104918

[CR33] Khare S, Khare T, Ramanathan R, Ibdah JA (2022) Hepatocellular carcinoma: the role of microRNAs. Biomolecules 12(5):645. 10.3390/biom12050645 35625573 10.3390/biom12050645 PMC9138333

[CR34] Kong M, Tian S, Shi H et al (2012) The effect of alpha-fetoprotein on the activation and phagocytosis of granulocytes and monocytes. Hepatogastroenterology 59(120):2385–2388. 10.5754/hge12296 22709874 10.5754/hge12296

[CR35] Krenkel O, Tacke F (2017) Liver macrophages in tissue homeostasis and disease. Nat Rev Immunol 17:306–321. 10.1038/nri.2017.11 28317925 10.1038/nri.2017.11

[CR36] Krenkel O, Puengel T, Govaere O et al (2018) Therapeutic inhibition of inflammatory monocyte recruitment reduces steatohepatitis and liver fibrosis. Hepatology 67(4):1270–1283. 10.1002/hep.29544 28940700 10.1002/hep.29544

[CR37] Lo Surdo P, Iannuccelli M, Contino S et al (2023) SIGNOR 3.0, the SIGnaling network open resource 3.0: 2022 update. Nucleic Acids Res 51(D1):D631–D637. 10.1093/nar/gkac883 36243968 10.1093/nar/gkac883 PMC9825604

[CR38] Lu Y, Chan YT, Tan HY et al (2022) Epigenetic regulation of ferroptosis via ETS1/miR-23a-3p/ACSL4 axis mediates sorafenib resistance in human hepatocellular carcinoma. J Exp Clin Cancer Res 41(1):3. 10.1186/s13046-021-02208-x 34980204 10.1186/s13046-021-02208-x PMC8722264

[CR39] Madhyastha R, Madhyastha H, Nurrahmah QI et al (2021) MicroRNA 21 elicits a pro-inflammatory response in macrophages, with exosomes functioning as delivery vehicles. Inflammation 44:1274–1287. 10.1007/s10753-021-01415-0 33501624 10.1007/s10753-021-01415-0

[CR40] Mahesh G, Biswas R (2019) MicroRNA-155: a master regulator of inflammation. J Interferon Cytokine Res 39(6):321–330. 10.1089/jir.2018.0155 30998423 10.1089/jir.2018.0155 PMC6591773

[CR41] Maini AA, Becares N, China L et al (2021) Monocyte dysfunction in decompensated cirrhosis is mediated by the prostaglandin E2-EP4 pathway. JHEP Rep 3(6):100332. 10.1016/j.jhepr.2021.100332 34825153 10.1016/j.jhepr.2021.100332 PMC8603213

[CR42] Martín-Sierra C, Martins R, Coucelo M et al (2020) Elevated soluble TNFα levels and upregulated TNFα mRNA expression in purified peripheral blood monocyte subsets associated with high-grade hepatocellular carcinoma. J Inflamm 17(1):1–11. 10.1186/s12950-020-00243-7 10.1186/s12950-020-00243-7 PMC7106708 32256215

[CR43] Mattoscio D, Isopi E, Lamolinara A et al (2021) Resolvin D1 reduces cancer growth stimulating a protective neutrophil-dependent recruitment of anti-tumor monocytes. J Exp Clin Cancer Res 40(1):1–16. 10.1186/s13046-021-01937-3 33845864 10.1186/s13046-021-01937-3 PMC8040222

[CR44] Melino M, Gadd VL, Alexander KA et al (2016) Spatiotemporal characterization of the cellular and molecular contributors to liver fibrosis in a murine hepatotoxic-injury model. Am J Pathol 186(3):524–538. 10.1016/j.ajpath.2015.10.029 26762581 10.1016/j.ajpath.2015.10.029

[CR45] Mohamed AA, Omar AAA, El-Awady RR et al (2020) Hsa-miR-155-5p and miR-665 role as potential non-invasive biomarkers for hepatocellular carcinoma in Egyptian patients with chronic hepatitis C virus infection. J Transl Int Med 8(1):32–40. 10.2478/jtim-2020-0006 32435610 10.2478/jtim-2020-0006 PMC7227164

[CR46] Munson PV, Adamik J, Butterfield LH (2022) Immunomodulatory impact of α-fetoprotein. Trends Immunol 43(6):438–448. 10.1016/j.it.2022.04.001 35550875 10.1016/j.it.2022.04.001

[CR47] Ong SM, Teng K, Newell E et al (2019) A novel five-marker alternative to CD16–CD14 gating to identify the three human monocyte subsets. Front Immunol 10:1761. 10.3389/fimmu.2019.0176 31402918 10.3389/fimmu.2019.01761 PMC6676221

[CR48] Patel VK, Williams H, Li SCH et al (2017) Monocyte inflammatory profile is specific for individuals and associated with altered blood lipid levels. Atherosclerosis 263:15–23. 10.1016/j.atherosclerosis.2017.05.026 28570862 10.1016/j.atherosclerosis.2017.05.026

[CR49] Riva A, Mehta G (2019) Regulation of monocyte-macrophage responses in cirrhosis-role of innate immune programming and checkpoint receptors. Front Immunol 10:167. 10.3389/fimmu.2019.00167 30804947 10.3389/fimmu.2019.00167 PMC6370706

[CR50] Rogacev KS, Zawada AM, Emrich I et al (2014) Lower Apo A-I and lower HDL-C levels are associated with higher intermediate CD14++CD16+ monocyte counts that predict cardiovascular events in chronic kidney disease. Arterioscler Thromb Vasc Biol 34(9):2120–2127. 10.1161/atvbaha.114.304172 25060791 10.1161/ATVBAHA.114.304172

[CR51] Ruiz-Alcaraz AJ, Tapia-Abellán A, Fernández-Fernández MD et al (2016) A novel CD14 (high) CD16 (high) subset of peritoneal macrophages from cirrhotic patients is associated to an increased response to LPS. Mol Immunol 72:28–36. 10.1016/j.molimm.2016.02.012 26938502 10.1016/j.molimm.2016.02.012

[CR52] Scarpellini E, Tack J (2012) Obesity and metabolic syndrome: an inflammatory condition. Dig Dis 30:148–153. 10.1159/000336664 22722429 10.1159/000336664

[CR53] Segarra G, Cortina B, Mauricio MD et al (2016) Effects of asymmetric dimethylarginine on renal arteries in portal hypertension and cirrhosis. World J Gastroenterol 22(48):10545. 10.3748/wjg.v22.i48.10545 28082806 10.3748/wjg.v22.i48.10545 PMC5192265

[CR54] Shi X, Yu Z, Ren P et al (2023) HUSCH: an integrated single-cell transcriptome atlas for human tissue gene expression visualization and analyses. Nucleic Acids Res 51(D1):D029-D1037. 10.1093/nar/gkac1001 10.1093/nar/gkac1001 PMC9825509 36318258

[CR55] Shikuma CM, Chow DC, Gangcuangco LM et al (2014) Monocytes expand with immune dysregulation and is associated with insulin resistance in older individuals with chronic HIV. PLoS ONE 9(2):e90330. 10.1371/journal.pone.0090330 24587328 10.1371/journal.pone.0090330 PMC3937368

[CR56] Shu X, Chen XX, Kang XD et al (2022) Identification of potential key molecules and signaling pathways for psoriasis based on weighted gene co-expression network analysis. World J Clin Cases 10(18):5965–5983. 10.12998/wjcc.v10.i18.5965 35949853 10.12998/wjcc.v10.i18.5965 PMC9254198

[CR57] Tellapuri S, Sutphin PD, Beg MS et al (2018) Staging systems of hepatocellular carcinoma: a review. Indian J Gastroenterol 37(6):481–491. 10.1007/s12664-018-0915-0 30593649 10.1007/s12664-018-0915-0

[CR58] Tsoris A, Marlar CA (2023) Use of the Child Pugh score in liver disease. StatPearls, Treasure Island 31194448

[CR59] Verstegen MM, Pan Q, van der Laan LJ (2015) Gene therapies for hepatitis C virus. Adv Exp Med Biol 848:1–29. 10.1007/978-1-4939-2432-5_1 25757613 10.1007/978-1-4939-2432-5_1

[CR60] Wang X, Wang Q (2018) Alpha-fetoprotein and hepatocellular carcinoma immunity. Can J Gastroenterol Hepatol 2018:9049252. 10.1155/2018/9049252 29805966 10.1155/2018/9049252 PMC5899840

[CR61] Wei L, Wang X, Lv L et al (2019) The emerging role of microRNAs and long noncoding RNAs in drug resistance of hepatocellular carcinoma. Mol Cancer 18(1):147. 10.1186/s12943-019-1086-z 31651347 10.1186/s12943-019-1086-z PMC6814027

[CR62] Wolf AA, Yáñez A, Barman PK, Goodridge HS (2019) The ontogeny of monocyte subsets. Front Immunol 10:1642. 10.3389/fimmu.2019.01642 31379841 10.3389/fimmu.2019.01642 PMC6650567

[CR63] Wong KL, Tai JJ, Wong WC et al (2011) Gene expression profiling reveals the defining features of the classical, intermediate, and nonclassical human monocyte subsets. Blood 118(5):e16-31. 10.1182/blood-2010-12-326355 21653326 10.1182/blood-2010-12-326355

[CR64] Yilma M, Saxena V, Mehta N (2022) Models to predict development or recurence of hepatocellular carcinoma (HCC) in patients with advanced hepatic fibrosis. Curr Gastroenterol Rep 24(1):1–9. 10.1007/s11894-022-00835-8 35142988 10.1007/s11894-022-00835-8 PMC8891098

[CR65] Yousef MH, El-Fawal HAN, Abdelnaser A (2020) Hepigenetics: a review of epigenetic modulators and potential therapies in hepatocellular carcinoma. BioMed Res Int 2020:9593254. 10.1155/2020/9593254 33299889 10.1155/2020/9593254 PMC7707949

[CR66] Youssef SS, Hamdy NM (2017) SOCS1 and pattern recognition receptors: TLR9 and RIG-I; novel haplotype associations in Egyptian fibrotic/cirrhotic patients with HCV genotype 4. Arch Virol 162:3347–3354. 10.1007/s00705-017-3498-7 28762092 10.1007/s00705-017-3498-7

[CR67] Zhang J, Li D, Zhang R et al (2020) The miR-21 potential of serving as a biomarker for liver diseases in clinical practice. Biochem Soc Trans 48(5):2295–2305. 10.1002/mc.20712 33119045 10.1042/BST20200653

[CR68] Zhu X, Zhou H (2022) Neutrophil-to-lymphocyte ratio can distinguish patients with liver cirrhosis from healthy people but cannot distinguish patients with cirrhotic hepatocellular carcinoma from patients with liver cirrhosis. J Hepatocell Carcinoma 9:1127–1136. 10.2147/JHC.S387189 36338430 10.2147/JHC.S387189 PMC9628700

