# Supplementary material for: Monocytes subsets altered distribution and dysregulated plasma hsa-miR-21-5p and hsa-miR-155-5p in HCV-linked liver cirrhosis progression to hepatocellular carcinoma
Source: J Cancer Res Clin Oncol. 2023 Aug 28;149(17):15349–64. doi: 10.1007/s00432-023-05313-w (PMC10620275; doi:10.1007/s00432-023-05313-w)
Supplement: Supplementary file 1 — Supplementary file1 (DOCX 31 KB) [file 432_2023_5313_MOESM1_ESM.docx]

| **Supplementary Table S1** Monocytes subsets frequencies & miRs in LC patients without HCC (n= 40) and with HCC (n=39) according to child score | | | |
| --- | --- | --- | --- |
| **Group, n** | **LC group without HCC, 40, child score** | | ***P* value** |
| **Characteristics (unit)** | **A, 12** | **B & C, 28** |  |
| **Total monocytes %** | 6.9 (4.8 - 7.9) | 5.7 (4.9 - 7.3) | NS |
| **Classical monocytes %** | 4.5 (3.9 - 5.5) | 3.8 (3.2 - 5.86) | NS |
| **Intermediate monocytes %** | 0.68 (0.37 - 1.4) | 0.65 (0.50 - 0.98) | NS |
| **Non-classical monocytes %** | 0.63 (0.34 - 2.0) | 0.43 (0.21 -0.70) | NS |
| **hsa-miR-21-5p fold changes** | **10.7(9.7 - 15.1)** | **4.9 (2.9 - 10.8)** | **0.009*** |
| **hsa-miR-155-5p fold changes** | 1.9 (1.3 - 2.3) | 1.4 (0.5 - 2.2) | NS |
| **Group, n** | **LC with HCC group, 39 child score** | |  |
| **Characteristics (unit)** | **A, 24** | **B & C, 15** | ***P* value** |
| **Total monocytes %** | 6.7(5.0 -8.7) | 7.3(5.1 -11.0) | NS |
| **Classical monocytes %** | 4.8(3.4 -6.4) | 4.4(3.5 -8.5) | NS |
| **Intermediate monocytes %** | 1.1(0.79 -1.9) | 1.19(1.0 -1.9) | NS |
| **Non-classical monocytes %** | 0.43(0.20 -.87) | 0.69(0.19 -.91) | NS |
| **hsa-miR-21-5p fold changes** | 29.5(16.0 -64.6) | 24.4(6.5 -76.6) | NS |
| **hsa-miR-155-5p fold changes** | 4.7(1.7-10.9) | 1.9(1.5 -5.8) | NS |
| Data are median (inter quartile range (1^st^-3^rd^ quartile)), statistics were computed using SPSS software,  * statistical significance *p*-value < 0.05, NS, non-significant. | | | |

| **Supplementary Table S2** Spearman's correlation coefficient among investigated frequency of monocytes subsets in all post-HCV patients (n= 79) | | | | | | | | |
| --- | --- | --- | --- | --- | --- | --- | --- | --- |
|  | **post-HCV G4 patients (n= 79)** | | | | | | | |
| **Monocytes %** | **Total** | | **Classical** | | **Intermediate** | | **Non-classical** | |
| **Characteristics** | *r* | ***p*-value** | *r* | ***p*-value** | *r* | ***p*-value** | *r* | ***p*-value** |
| **Age (years)** | 0.051 | 0.656 | 0.043 | 0.705 | 0.132 | 0.248 | 0.031 | 0.784 |
| **BMI (kg/m^2^)** | -0.077 | 0.500 | -0.055 | 0.629 | -0.044 | 0.700 | -0.064 | 0.573 |
| **s. Insulin (mIU/L)** | -0.066 | 0.621 | -0.151 | 0.254 | 0.067 | 0.612 | -0.210 | 0.110 |
| **Insulin resistance** | 0.015 | 0.910 | -0.012 | 0.928 | **0.266** | **0.042** | -0.002 | 0.988 |
| **AFP (ng/mL)** | 0.106 | 0.352 | 0.078 | 0.492 | **0.258** | **0.022** | 0.044 | 0.700 |
| **AST (U/L)** | 0.029 | 0.803 | -0.075 | 0.511 | **0.224** | **0.047** | -0.081 | 0.480 |
| **ALT (U/L)** | -0.112 | 0.326 | -0.133 | 0.241 | -0.076 | 0.507 | -0.142 | 0.211 |
| **ALP (U/L)** | 0.030 | 0.791 | -0.019 | 0.869 | 0.025 | 0.829 | -0.049 | 0.669 |
| **GGT (U/L)** | -0.062 | 0.587 | -0.103 | 0.368 | -0.030 | 0.792 | -0.107 | 0.349 |
| **TAG (mg/dL)** | -0.078 | 0.493 | 0.004 | 0.969 | 0.087 | 0.447 | -0.159 | 0.162 |
| **TC (mg/dL)** | -0.133 | 0.242 | -0.085 | 0.459 | 0.046 | 0.686 | 0.049 | 0.671 |
| **HDL-C (mg/dL)** | -0.059 | 0.606 | .000 | 0.999 | **-0.225** | **0.046** | 0.191 | 0.092 |
| **TAG/HDL-C** | -0.024 | 0.835 | 0.046 | .686 | 0.129 | 0.256 | -0.205 | 0.070 |
| **NLR** | 0.093 | 0.414 | 0.138 | 0.225 | -0.010 | 0.928 | 0.098 | 0.392 |
| **PLR** | -0.054 | 0.634 | -0.049 | 0.669 | 0.019 | 0.869 | 0.057 | 0.615 |
| **LMR** | 0.154 | 0.174 | 0.207 | 0.067 | 0.048 | 0.675 | 0.065 | 0.569 |
| **hsa-miR-21-5p** | 0.066 | 0.565 | 0.051 | 0.654 | **0.300** | **0.007*** | -0.195 | 0.085 |
| **hsa-miR-155-5p** | 0.093 | 0.415 | 0.013 | 0.909 | 0.181 | 0.109 | **-0.316** | **0.005*** |
| **Number of liver masses** | 0.153 | 0.352 | 0.126 | 0.445 | -0.004 | 0.981 | 0.271 | 0.096 |
| Spearman correlation coefficient (r) was calculated using SPSS software, * significant correlation at p<0.05 level (2-tailed), NS; nonsignificant, # analyzed by point-biserial correlation. [ALT, alanine aminotransferase; AST, aspartate aminotransferase, AFP, alpha feto protein, BMI, Body mass index; HDL, high-density lipoprotein; GGT, gamma glutamyl transferase; LC, liver cirrhosis; PLR, platelet lymphocyte ratio; NLR, neutrophil lymphocyte ratio; LMR, lymphocyte monocyte ratio; TG, triglycerides.] | | | | | | | | |

| **Supplementary Table S3** Spearman's correlation coefficient among inflammatory ratios in all post-HCV patients (n= 79) | | | | | | | | |
| --- | --- | --- | --- | --- | --- | --- | --- | --- |
| **Group, n** | **post-HCV G4 patients, 79** | | | | | | | |
| **Inflammatory ratios** | **AMC** | | **LMR** | | **NLR** | | **PLR** | |
| **Characteristics (unit)** | *r* | ***p*-value** | *r* | ***p*-value** | *r* | ***p*-value** | *r* | ***p*-value** |
| **Age (years)** | 0.071 | 0.533 | -0.098 | 0.390 | 0.077 | 0.500 | 0.094 | 0.409 |
| **BMI (kg/m^2^)** | -0.148 | 0.193 | -0.019 | 0.865 | 0.050 | 0.664 | -0.089 | 0.433 |
| **s. Insulin (mIU/L)** | 0.111 | 0.402 | -0.082 | 0.539 | 0.151 | 0.254 | 0.115 | 0.387 |
| **Insulin resistance** | 0.042 | 0.751 | -0.108 | 0.414 | 0.011 | 0.934 | 0.184 | 0.162 |
| **AFP (ng/mL)** | -0.124 | 0.278 | 0.163 | 0.1052 | 0.063 | 0.579 | 0.035 | 0.762 |
| **AST (U/L)** | -0.146 | 0.201 | -0.102 | 0.372 | 0.013 | 0.912 | 0.105 | 0.359 |
| **ALT (U/L)** | -0.116 | 0.309 | -0.017 | 0.882 | -0.041 | 0.722 | 0.039 | 0.735 |
| **ALP (U/L)** | 0.031 | 0.788 | -0.104 | 0.360 | -0.119 | 0.295 | 0.181 | 0.110 |
| **GGT (U/L)** | -0.097 | 0.395 | -0.020 | 0.862 | -0.108 | 0.345 | 0.182 | 0.109 |
| **TAG (mg/dL)** | -0.079 | 0.486 | 0.024 | 0.836 | 0.156 | 0.171 | -0.035 | 0.759 |
| **TC (mg/dL)** | -0.093 | 0.417 | 0.065 | 0.569 | 0.189 | 0.096 | -0.155 | 0.173 |
| **HDL-C (mg/dL)** | -0.095 | 0.406 | 0.139 | 0.222 | -0.021 | 0.854 | -0.134 | 0.238 |
| **TAG/HDL-C** | 0.009 | 0.937 | -0.038 | 0.739 | 0.106 | 0.354 | 0.025 | 0.824 |
| **hsa-miR-21-5p** | -0.095 | 0.407 | 0.047 | 0.680 | 0.139 | 0.222 | -0.045 | 0.693 |
| **hsa-miR-155-5p** | **-0.233** | **0.039*** | 0.024 | 0.837 | 0.079 | 0.488 | 0.137 | 0.230 |
| **Number of liver masses** | -0.252 | 0.121 | 0.089 | 0.589 | 0.089 | 0.589 | 0.016 | 0.925 |
| Spearman correlation coefficient (r) was calculated using SPSS software, * significant correlation at p<0.05 level (2-tailed), NS; nonsignificant, # analyzed by point-biserial correlation. [ALT, alanine aminotransferase; AST, aspartate aminotransferase, AFP, alpha feto protein, BMI, Body mass index; HDL, high-density lipoprotein; GGT, gamma glutamyl transferase; LC, liver cirrhosis; PLR, platelet lymphocyte ratio; NLR, neutrophil lymphocyte ratio; LMR, lymphocyte monocyte ratio; TG, triglycerides.] | | | | | | | | |
